# Genetic Characterization of Fungal Biodiversity in Storage Grains: Towards Enhancing Food Safety in Northern Uganda

**DOI:** 10.3390/microorganisms9020383

**Published:** 2021-02-14

**Authors:** Godfrey Wokorach, Sofie Landschoot, Kris Audenaert, Richard Echodu, Geert Haesaert

**Affiliations:** 1 Department of Plants and Crops, Campus Schoonmeersen Building C, Faculty of Bioscience Engineering, Ghent University, Valentin Vaerwyckweg 1, B-9000 Ghent, Belgium; Sofie.Landschoot@UGent.be (S.L.); Kris.Audenaert@UGent.be (K.A.); Geert.Haesaert@UGent.be (G.H.); 2 Multifunctional Research Laboratory, Gulu University, P.O. Box 166, Gulu, Uganda; richardechodu2009@gmail.com; 3 Department of Biology, Faculty of Science, Gulu University, P.O. Box 166, Gulu, Uganda

**Keywords:** *Fusarium*, Uganda, fumonisins, *Curvularia*

## Abstract

Worldwide fungal contamination leads to both quantitative and qualitative grain losses during crop growth and/or storage. A greater proportion of grains contamination with toxins often occurs in sub-Saharan Africa, where control measures are limited. We determined fungal diversity and their toxin production ability in household grains meant for human consumption to highlight the risk of mycotoxin exposure among people from northern Uganda. The study underlines the high diversity of fungi that group into 15 genera; many of which are plant pathogens with toxigenic potential. *Fusarium verticillioides* was the most common fungal species isolated from household grains. The study also indicates that northern Uganda is favored by a high proportion of toxigenic isolates of *F. verticillioides*, *F. andiyazi*, and *F. proliferatum*, which are characterized by a high fumonisins production capability. The fumonisins production ability was not dependent on the species, grain types, and haplotype group to which the isolates belong. The contamination of most household grains with fungi capable of producing a high amount of toxin shows that most people are exposed to an elevated amount of mycotoxins, which shows the frequent problems with mycotoxins that have been reported in most parts of sub-Saharan Africa.

## 1. Introduction

Crops are susceptible to infections by a variety of plant pathogens [1], of which fungi are the most widespread and destructive, infecting crops in the field and during storage [2,3]. Fungi are responsible for a wide range of serious plant diseases. Ear rot, mainly caused by *Fusarium* and *Aspergillus* species, is one example of a disease that constrains the production of cereals worldwide [4]. Besides crop losses, some fungal infections of field crops result in contamination of food/feed with mycotoxins during crop growth or storage [5]. Most field fungi continue to proliferate and multiply during storage owing to inappropriate storage conditions. In sub-Saharan Africa, the storage of grains is unavoidable to maintain a constant supply given the periodic production pattern [6]. Storage of grains in many rural African settings, typical of northern Uganda, comes with an elevated cost of grains contamination with fungi due to inappropriate storage structure [7]. The common grain storage structure comprises; sisal bags, pits, wood and wire cribs, wooden open-air, polythene bags, and heaping on floor [8]. Such a storage structure exposes grains to high humidity, storage pests (insects, mites, birds, rodents), and fungal contamination [9],⁠ which increases fungal diversity, proliferation, and toxin levels in stored cereal grains. Spoilage of grains with fungal toxins appears to increase when grains take longer within storage facilities in African settings [10]. As such, most grains produced in sub-Saharan Africa are often contaminated with mycotoxins besides severe yield losses caused by a fungal infection [11,12,13]. In east Africa, fatality due to dietary consumption of grains or grain-based products contaminated with aflatoxins is the highest in the world, ranging between 29% and 33% [14,15,16]. The overall damage to field crops and grains largely depends on the climate and prevailing weather conditions, which also influence the diversity and distribution of the fungi [17,18]. As such, the warm and humid conditions of northern Uganda would provide an ideal environment for the proliferation and multiplication of diverse fungal species.

Furthermore, global trade of agricultural products and trans-boundary traffic [1] contribute to the spread and distribution of diverse pathogenic and toxigenic fungal species. The penultimate consequence of fungal infection of crops and contamination of grains with toxins in sub-Sahara Africa are hunger, malnutrition, financial losses, poor health, and death of persons which need to be sorted urgently. Worldwide, several species of toxigenic and pathogenic fungi have been characterized. The characterization of fungal diversity and their distribution among household grains in Uganda is a worthwhile undertaking to improve food safety, given the high level of mycotoxins contamination of grains in Uganda [13,19,20]. The limited knowledge on the occurrence and distribution pattern of the different fungi in Uganda make the current crop protection and food safety regulation procedures fall short of many unidentified fungal pathogens, which may present a silent burden to farmers in terms of toxin contaminations of food and crop damage [21]. A deeper understanding of the fungal population and the influence of the environment and other factors is important for the implementation of good food safety measures in Uganda. Therefore, this study aimed at identifying fungi associated with storage grains in northern Uganda to help mitigate widespread crop losses and mycotoxins contamination of grains. In this study, we adopt molecular identification based on DNA sequencing of a partial fragment of internal transcribed spacer region (ITS) and translation elongation factor alpha1 (TEF-α1) gene [22,23,24] to gain insight into fungal genera and species associated with grains in northern Uganda. The translation elongation factor alpha1 genes have been proven to offer a good resolution to distinguish between fungal genera and species [22,25]. From the partial TEF-α1 nucleotide sequence, we determined the fungal species diversity associated with grains in northern Uganda, and measured genetic differentiation among isolates from Uganda and species reported in other countries to determine evidence of gene flow into Uganda. The potential of the fungi to produce mycotoxins was evaluated by PCR amplification and sequencing of the core genes involved in the toxin-production pathways. The ability of the fungi to produce mycotoxins was evaluated on growth media and quantified using the ELISA-method. Produced mycotoxin concentrations were compared to determine species associated with high mycotoxin production potential.

## 2. Materials and Methods

### 2.1. Description of Study Area

The study area was in the northern part of Uganda, bordered by South Sudan and the Democratic Republic of Congo (DRC). The total land area of northern Uganda is 85,391.7 km^2^, and most of the 7,188,139 people depend on agriculture [26]. The grains were collected from nine districts (Gulu, Nwoya, Kole, Oyam, Omoro, Lira, Kitgum, Pader, and Lamwo). A greater percentage of the farming population in northern Uganda are peasants who practice subsistence agriculture for livelihood requirements [27]. The major crops that support the calorie requirement of people of northern Uganda are maize, sorghum, millet, and groundnut [28]. The climate is structured in two seasons that determine the agricultural cycles of northern Uganda. The hot and dry season usually lasts from December to February due to dry air currents that come from the arid regions of Sudan and Eritrea. The dry season is marked with no or little precipitation and less active growing of crops. The rainy season starts in April and ends in October, it is less hot, but more humid due to moist air currents that come from DR Congo. The rainy season is characterized by high precipitation and the active growing of crops.

### 2.2. Sample Collection

The grains that were intended for domestic households’ consumption were collected from 380 different households spread across the nine districts in northern Uganda. The grains considered were from the five major cereal crops that are commonly grown in northern Uganda (maize, rice, sorghum, and millet) and peanut and sesame commonly used as a paste. From each household, approximately half a kilogram of each grain type/legume was collected. The samples were transferred into clean Ziploc and then shield. The Ziploc bag was labeled with a unique sample identifier code. All the samples were transported to Gulu University Multi-functional Research Laboratories for fungal isolation. The fungal isolates (*n* = 160) analyzed were distributed among the nine districts as follows: Gulu (*n* = 41), Nwoya (*n* = 37), Kole (*n* = 13), Oyam (*n* = 4), Omoro (*n* = 19), Lira (*n* = 22), Kitgum (*n* = 7), Pader (*n* = 12), and Lamwo (*n* = 5).

### 2.3. Isolation of Fungi from Grains

From each sample, five grains selected at random were disinfected with 70% alcohol for 3 min and rinsed with distilled water twice. Grains were transferred to Petri dishes containing Potato Dextrose Agar (PDA, 40 g/L) that was supplemented with penicillin V to prevent bacterial growth. The Petri dishes were incubated at room temperature until the fungal growth started. Petri dishes with no fungal growth were categorized as grains with no fungal contamination. Sub-culturing was done on a new PDA medium supplemented with penicillin V. The fungal isolates were transferred to liquid Potato Dextrose Broth (PDB, 24 g/L) and grown for 10 days to develop dense mycelium. The mycelium was harvested into 2 mL Eppendorf tubes and centrifuged at 12,000 rpm for two minutes. The supernatant, which largely contains the broth, was discarded, and the mass of mycelium pellet was kept. The mycelium pellet was frozen for 10 min at −80 °C and was lyophilized for 12 h.

### 2.4. DNA Extraction

The lyophilized mycelia were frozen in liquid nitrogen and ground into a powder with a micro pestle inside the 2 mL tubes. A total of 500 µL of CTAB buffer containing 1% of proteinase K enzyme was added to a 2 mL tube containing powder-like ground mycelium and mixed by brief vortexing. The mixture was incubated for one hour in a water bath set at 65 °C. Then 500 µL of chloroform:isoamyl alcohol (3:1) was added to the homogenate and incubated for 10 min. The mixture was centrifuged at 13,000 rpm for five minutes. From the upper aqueous phase; 300 µL were transferred into new 2 mL tubes. To precipitate the DNA, 300 µL of pre-chilled isopropanol kept at −20 °C was added and then mixed by inverting the tube slowly five times. The mixture was again centrifuged at 13,000 rpm for 15 min to pellet the DNA precipitate. The supernatant was discarded, and the pellet washed with 200 µL of 70% ethanol followed by centrifugation at 13,000 rpm for 2 min. The ethanol supernatant was again discarded, and the pellet was allowed to dry for 15 min. The dry pellet was dissolved in 50 µL of elution buffer.

### 2.5. PCR Reaction and Sequencing

For the PCR reaction, the primer set ITS4 (5′-TCC TCCGCTTATTGATATGC-3′) and ITS5 (5′-GGAAGTAAAAGTCGTAACAAGG-3′) designed from internal transcribed spacer region was used for classification of the fungal genera. Primer set EF1 (5′-ATGGGTAAGGARGACAAGAC-3′) and EF2 (5′-GGARGTACCAGTSATCATGTT-3′) that amplify part of translation elongation factor alpha1 gene was used for the classification of species [29]. Primers rp32 (5′-ACAAGTGTCCTTGGGGTCCAGG-3′) and rp33 (5′GATGCTCTTGGAAGTGGCCTACG-3′) were used for the detection of the FUM1 gene among *Fusarium* species [30]. Lastly, primer set 72F (5′-TACAGCTCTGGACGAAGTGTTCC-3′) and 726R (5′-GGCGCAGAAAGTAACAGCCA-3′) was used for the detection of the FUM3 gene among *Fusarium* species.

The PCR was run using the following composition of reagents: 5 µL GoTag PCR-buffer (5X), 1.25 µL dNTP (10 mM) solution mix, 1 µL (5 nM) forward primer, 1 µL (5 nM) reverse primer, 0.125 µL of GoTaq polymerase (5 U/µL), 2 µL DNA template, and 14.625 µL nuclease-free water. Amplifications were done using the following temperature conditions: 1 cycle of initial denaturation at 94 °C for 3 min; 35 cycles of actual denaturation at 94 °C for 1 min, primer annealing at 52 °C for 30 s, and extension at 72 °C for 45 s. Final extension at 72 °C for 5 min and reaction hold at 4 °C. The quality of the PCR product was evaluated on 1% agarose gel. The amplicon was purified using MicroElute^®^ Cycle-Pure Kit (www.omegabiotek.com). The concentration of the amplicons was measured with a fluorometer and the concentration adjusted to the range of 10–30 ng/µL. The amplicons were sequenced using Sanger technology by LGC Genomics GmbH (Isabel Czeski, Ostendstrasse 25, TGS Haus 8, and 12,459 Berlin, Germany).

### 2.6. Sequence Processing and Analysis

The quality of the chromatograms was inspected in Mega X. Quality trimming was done at the beginning and the end of the sequence to remove sequencing primers and poor quality nucleotide base calls. Using the basic GenBank local alignment search tool program (Mega BLAST), the collected nucleotide sequences were searched against the NCBI database [31] to identify the collected fungi (high identity score and low e-value). Highly similar sequences present in the NCBI database were downloaded and used for comparative analysis against the sequences of isolates from this study (Supplementary Tables S1 and S2).

### 2.7. Phylogenetic Tree Construction

Multiple alignments were done using the ClustalW algorithm with a Gap Opening Penalty of 15.00 and Gap Extension Penalty of 6.66 in MegaX [32]. The best DNA/Protein model for the phylogenetic tree was selected based on the Automatic (neighbor-joining tree) and the maximum likelihood method of nucleotides substitution. The maximum likelihood phylogenetic trees were constructed in MegaX with a bootstrap value of 1050 replications.

### 2.8. Genetic Differentiation of the Fungi Using TEF-α1 Gene

Genetic differentiation for the different fungal species isolated from northern Uganda was analyzed using the TEF-α1 gene nucleotide encoding sequence, as it is considered highly informative among *Fusarium* [22]. Fixation indices (*Fst*) were used to determine genetic differentiation and gene flow between different populations and geographical regions. *Fst* values were defined as follows: Little genetic differentiation (*Fst* = 0–0.05), which indicate evidence of gene flow, moderate genetic differentiation (*Fst* = 0.05–0.15), great genetic differentiation (*Fst* = 0.15–0.25), and very great genetic differentiation (*Fst* > 0.25) [33]. The Fixation indices were determined between isolates derived from Uganda and those reported in other countries.

### 2.9. Haplotype Clustering

Population diversity was described by the number of haplotype groups using the nucleotide sequence of the TEF-α1 gene [34]. The haplotype was defined as a set of single nucleotide polymorphisms (SNPs) that occur in particular isolates or sub-groups and were calculated using DnaSP v.5 (University of Barcelona, Catalonia, Spain) [35,36]. The frequency of occurrence of the different haplotypes associated with the Uganda population and other countries based on TEF-α1 was determined and represented as bar charts. The diversity within the species was determined by the number of haplotype groups the isolates were able to form. The genetic relationship between Ugandan isolates and those derived from other countries was compared when the isolates from both countries cluster in the same haplotype group. The geographical distribution of haplotypes was classified as limited when the isolates of a given haplotype group only occur in Uganda or a particular country. The haplotype group was classified as having wider geographical distribution when the isolates that make up the haplotype group occur both in Uganda and other countries.

### 2.10. Detection of Fumonisin Gene Cluster among Fusarium Isolates

The genetic potential of the fungi to produce fumonisins was determined by amplifying and sequencing the FUM1 and FUM3 gene clusters. Isolates with amplification were recorded as having the gene. No amplification indicates the absence of the genes. The amplicons were clean and sequenced using Sanger sequencing technology. The sequences were searched against the NCBI database to confirm that the amplified sequences correspond to the fumonisins’ gene cluster.

### 2.11. Mycotoxins Extraction and Quantifications

The fungi were grown in PDA for 20 days. The content of the petri dish (mycelium + agar) was transferred into a bottle containing 50 mL of 70% methanol (70 mL methanol: 30 mL water) and vigorously shaken for three minutes. It was allowed to settle for five minutes and filtered with Whatman number 1 filter paper. Fumonisins concentration were quantified using an ELISA kit of Romer Labs^®^ Division Holding GmbH (Erber Campus 1, 3131 Getzersdorf, Austria). The AgraQuant^®^ fumonisin ELISA test kit used was designed for simultaneous quantitative analysis of fumonisins (B1, B2, and B3). The kit had a limit of detection of 0.20 mg/kg. The ELISA plate was read with a Multiskan ELISA reader with filters set at 630 nm and 450 nm wavelength. The results were calculated using the Romer Labs^®^ spreadsheet that uses the Log/Logit regression model to make a calibration curve with the absorbance reading and concentration of the standard solutions. The concentration of fumonisins produced by each isolate was calculated by interpolation from the calibration curve. Fumonisins concentrations below the limit of detection (LOD) were scored as negative, and those with detectable concentration were scored as positive.

### 2.12. Data Analysis

Prevalence of the different fungal genera and species occurring in grains was used to determine fungal species commonly infecting grains in northern Uganda. The prevalence per grain type was calculated as the number of fungal species/genera detected divided by the total number of fungal species/genera that were recovered and then multiplied by 100. Chi-square was used to determine whether genetic differentiation between the *Fusarium* isolates from Uganda and those isolates reported in other countries truly exists at a significant level of 0.05. Similarly, chi-square was used to test whether the proportion of *Fusarium* isolates with fumonisins gene clusters (FUM1 and FUM3) was more than isolates without fumonisins gene clusters at a significant level of 0.05. The normality of the fumonisins concentrations produced by the *Fusarium* species in media was tested using the Shapiro–Wilk test at a significance level of 0.05. Kruskal–Wallis test was used to test for the differences in fumonisins concentration produced by the three *Fusarium* species (*F. verticillioides*, *F. andiyazi*, and *F. proliferatum*) since the dataset did not meet the normality assumption. The level of significance was 5% (*p* < 0.05) for the statistical tests done. All the statistical analyses were done in R-Studio v4.0 (RStudio, PBC, Boston, MA, USA).

## 3. Results

### 3.1. Fungal Genera and Species Associated with Cereal Grains in Northern Uganda

A total of 160 isolates were amplified with ITS. The nucleotide sequences of the internal transcribed spacer region group the isolates into 15 fungal genera (Table 1). The majority of the isolates (*n* = 85, 51.25%) were of the genus *Fusarium*. *Bipolaris* was the second most common (*n* = 18, 11.25%) fungal genus associated with grains in Uganda. Among the isolates, 8.13% (*n* = 13) were classified as *Curvularia*. The proportion of isolates classified as *Epicoccum* was 7.50% (*n* = 12). Five percent (*n* = 8) of the isolates were a member of the genus *Lasiodiplodia*. Three of the isolates belonged to the genus *Macrophomina*. Some other genera were only marginally present (frequencies of less than 1%) (Table 1).

A total of *n* = 96 (60%) of the 160 isolates were amplified with TEF1-α primers for further identification, *n* = 64 (40%) isolates did not amplify for TEF1-α. The genera that did not amplify with TEF1-α were *Curvularia*, *Bipolaris*, *Epicoccum*, *Cladosporium*, and *Lasiodiplpdoa*. Characterization of the isolates using TEF1-α identified *Fusarium verticillioides* as the most common (*n* = 32, 20.00%) *Fusarium* species associated with grains in Uganda. *Fusarium incarnatum* was the second most frequent *Fusarium* species (*n* = 21, 13.13%) that was isolated from the grains. The proportions of *F. andiyazi* and *F. equiseti* were 11.25% and 6.88%, respectively (*n* = 18, *n* = 11). Other *Fusarium* species isolated were *F. thapsinum* (*n* = 2, 1.25%), *F. proliferatum* (*n* = 2, 1.25%), and *F. solani* (*n* = 4, 2.50%). *Trichoderma gamsii* (*n* = 1) and *Aspergillus flavus* (*n* = 1) only marginally occurred in the fungal population.

### 3.2. Distribution of the Fungi among the Different Grain Types

*Fusarium verticillioides* (13.04%) and *F. andiyazi* (7.45%) were the most common fungi isolated from maize grains. For sorghum grains, most of the fungi isolated were identified as *F. incarnatum* (4.97%) and *F. verticillioides* (3.11%). *F. incarnatum* (3.11%) was also the most common fungus isolated from millet grains, followed by *F. verticillioides* (1.24%) and *F. proliferatum* (1.24%). Note that *F. proliferatum* was only isolated from millet grains. *Fusarium equiseti* (3.73%) and *F. solani* (1.86%) were isolated from peanut. *F. solani* was only detected with minimal frequency in maize (0.62%). Similarly, F. thapsinum occurred at a minimal frequency in simsim (0.62%) and sorghum (0.62%) (Table 2).

### 3.3. Genetic Differentiation between Isolates from Uganda and Isolates Reported in Other Countries

The isolates were compared with those derived from other countries to determine the extent of genetic differentiation between Ugandan isolates and isolates reported in other countries. The study indicates strong evidence of high genetic differentiation among the *F. verticillioides* isolates from Uganda and isolates derived from other countries (Table 3). In addition, the genetic differentiation observed between isolates of *F. verticillioides* from this study (*n* = 29) and isolates from eastern Uganda (*n* = 20) obtained from the NCBI database was high (Fst = 0.11366, *p* = 0.0672). A similar trend was observed for genetic differentiation (Fst = 0.12586, *p* = 0.0029) for *F. verticillioides* isolates from the neighboring country, Kenya (Table 3). The observed genetic differentiation (Fst = 0.01764, *p* = 0.0853) between *F. verticillioides* from China and isolates from Uganda was significantly low. In contrast, the genetic differentiation of *Fusarium* isolates from most countries: Japan (Fst = 0.43, *p* = 0.0001), India (Fst = 0.21, *p* = 0.00), and the USA (Fst = 0.12078, *p* = 0.0013), with Ugandan isolates was high, which indicates evidence of limited gene flow between Uganda and these countries. Among the *F. andiyazi*, *F. equiseti*, and *F. incarnatum* isolates, none of the selected countries had evidence of gene flow with Ugandan isolates since a high genetic differentiation supported with a significant *p*-value for markers that define the differences among the isolates was observed (Table 3).

### 3.4. Haplotype Structure of Fusarium Species in Uganda Relative to Other Countries

**Figure 1:**
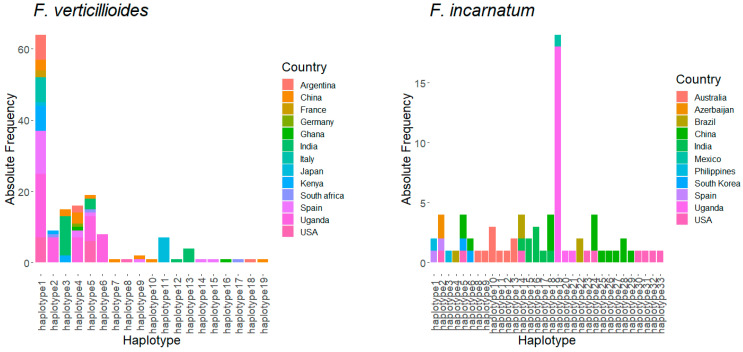

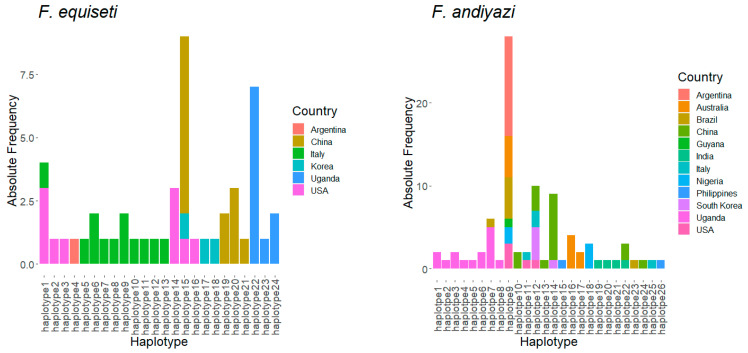
Distribution of different haplotypes derived from on partial sequence of translation elongation factor alpha-1 gene according to country of origin.

The evolutionary pattern of *Fusarium* species was determined by the haplotype structure, based on TEF1-α, of the *Fusarium* species isolated in Uganda relative to those from other countries. The result indicates that *F. verticillioides* isolates from Uganda (*n* = 47) were structured into five haplotype groups (Figure 1). Equally maximum likelihood phylogenetic tree clusters all the *F. verticillioides* isolates from Uganda into five clusters (Figure S1). Isolates from China (*n* = 13) and Spain (*n* = 18) had the highest number of haplotypes, 8 and 6, respectively, whereas all the isolates from Italy (*n* = 7) obtained from NCBI were homogenous and belonged to only one haplotype. Some haplotypes showed a limited geographical distribution within one or two countries only. For example, haplotype 6 and haplotype 2 of *F. verticillioides* showed a limited geographical distribution outside Uganda, whereas other haplotypes were found to occur in multiple countries (Figure 1 and Figure S2). The majority (*n* = 71) of the isolates of the *F. verticillioides* group were in haplotype 1, and it was the only haplotype group with the widest geographical distribution worldwide, including Uganda. Some countries that share haplotype 1 were: Uganda (*n* = 12), the USA (*n* = 7), Spain (*n* = 12), Kenya (*n* = 7), and China (*n* = 3).

For *F. andiyazi*, haplotype grouping indicated that the Ugandan isolates were different from isolates found in other countries. *F. andiyazi* isolates from Uganda were structured into eight different haplotype groups, and each of the haplotype groups had limited occurrence outside Uganda (Figure 1). In addition, the haplotypes of *F. andiyazi* for sequences derived from Brazil, China, and the USA show restricted distribution, just like what was observed for Ugandan isolates. Haplotype 9 of *Fusarium andiyazi* had a relatively wider geographical distribution as species belonging to this haplotype occur in Australia, Argentina, the USA, and Nigeria excluding Uganda. *Fusarium equiseti;* isolates originating from Italy (*n* = 12) showed the highest diversity, as they group into ten different haplotype groups compared with isolates derived from Uganda (*n* = 10) that were structured in only three haplotype groups. The three haplotype groups of *F. equiseti* (haplotype 22, haplotype 23, and haplotype 24) that occur in Uganda show limited distribution outside Uganda (Figure 1). Haplotype 22 of *F. equiseti* was the most common group associated with grain in Uganda, while the isolates belonging to haplotype groups (haplotype 23 and 24) were only marginally detected. The Ugandan isolates of *F. incarnatum* (*n* = 20) were also structured into three haplotypes (haplotype 19, 20, and 21). The majority of the isolates of *F. incarnatum* from Uganda belong to haplotype 19 (*n* = 18) while the other two haplotype groups (haplotype 20 and 21) were marginally represented. None of the *F. incarnatum* isolates reported in other countries were found to group with the three haplotype groups that occur in Uganda (Figure 1).

### 3.5. Detection of Fumonisins Gene Clusters (FUM1 and FUM3) among Fusarium Isolates

Fumonisins gene clusters (FUM1 and FUM3) were amplified and sequenced to determine fumonisins production potential of the isolates from northern Uganda. Both FUM1 and FUM3 genes were detected within the three *Fusarium* species (*F. verticillioides*, *F. andiyazi*, and *F. proliferatum*). The proportion of *F. verticillioides* isolates with the FUM1 gene (96.88%, *n* = 31) was significantly (X^2^ = 26.28, *p* = 2.95 × 10^−7^) higher than the proportion of isolates without the FUM1 gene (3.12%, *n* = 1) (Table 4). The same was observed for the FUM3 gene within the *F. verticillioides* population. All the isolates of *F. proliferatum* 100% (*n* = 2) were found to have both the FUM1 and FUM3 gene cluster. The difference in the proportion of isolates with FUM1 gene 61.11% (*n* = 11) and those without the FUM1 gene among *F. andiyazi* was not significant (X^2^ = 0.5, *p* = 0.47). The proportion of *F. andiyazi* with FUM3 gene 50% (*n* = 9) was equal to the proportion of isolates without the FUM3 gene (Table 4).

### 3.6. Genetic Variability within Fumonisin Gene Clusters (FUM1 and FUM3)

**Figure 2:**
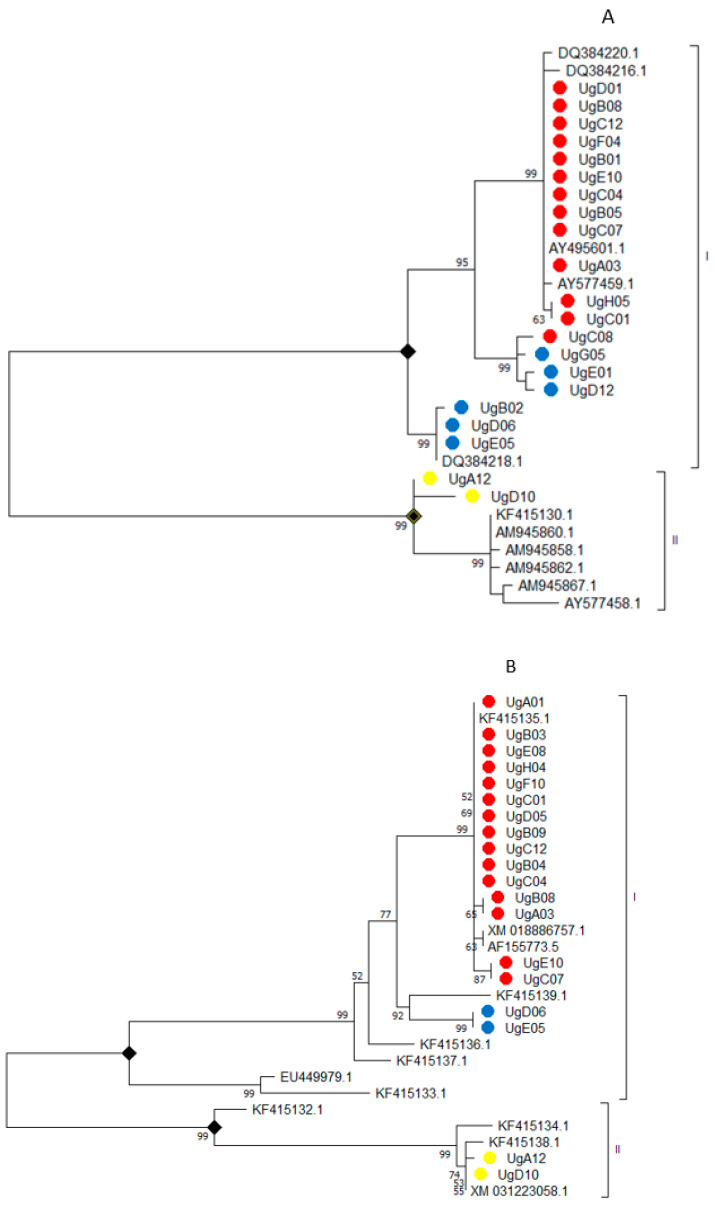
Phylogenetic tree of fumonisins gene cluster. **A** = FUM1 and **B** = FUM3. Yellow node (*F. proliferatum*), blue node (*F. andiyazi*), and red node (*F. verticillioides*).

The phylogenetic tree shows a divergence in the FUM1 gene among the *F. verticillioides*, *F. proliferatum*, and *F. andiyazi* isolates. Based on FUM1, both *F. verticillioides* and *F. andiyazi* appear to have a monophyletic origin that is distantly related to *F. proliferatum* (Figure 2A). Within the *F. verticillioides* and *F. andiyazi* main cluster (cluster I), there were three distinct sub-groups. The first subgroup had isolates only for *F. verticillioides*. The second subgroup had isolates from both *F. verticillioides* (UgC08) and *F. andiyazi* (UgG05, UgE01, and UgD12). The last subgroup within cluster I only had isolates of *F. andiyazi* (UgB02, UgD06, and UgE05). The genetic distance within the FUM1 gene between *F. verticillioides* and *F. andiyazi* (0.027) was lower than the genetic distance between *F. proliferatum* and *F. verticillioides* (0.153). Similarly, the genetic distance between *F. verticillioides* and *F. andiyazi* was lower than the observed genetic distance between *F. proliferatum* and *F. andiyazi* (0.149) within the FUM1 gene. Multiple alignments of FUM1 gene nucleotide sequences of isolates of both *F. verticillioides*, *F. andiyazi*, and *F. proliferatum* indicate deletion of four nucleotide fragments associated with *F. proliferatum* only (Figure S3). For the FUM3 gene, there was no clear separation between *F. verticillioides* and *F. andiyazi* (group I). However, isolates of *F. proliferatum* (UgA12 and UgD10) cluster together separately from *F. verticillioides* and *F. andiyazi isolates* (Figure 2B).

### 3.7. Mycotoxin Production Capability of the Fusarium Isolates

**Figure 3:**
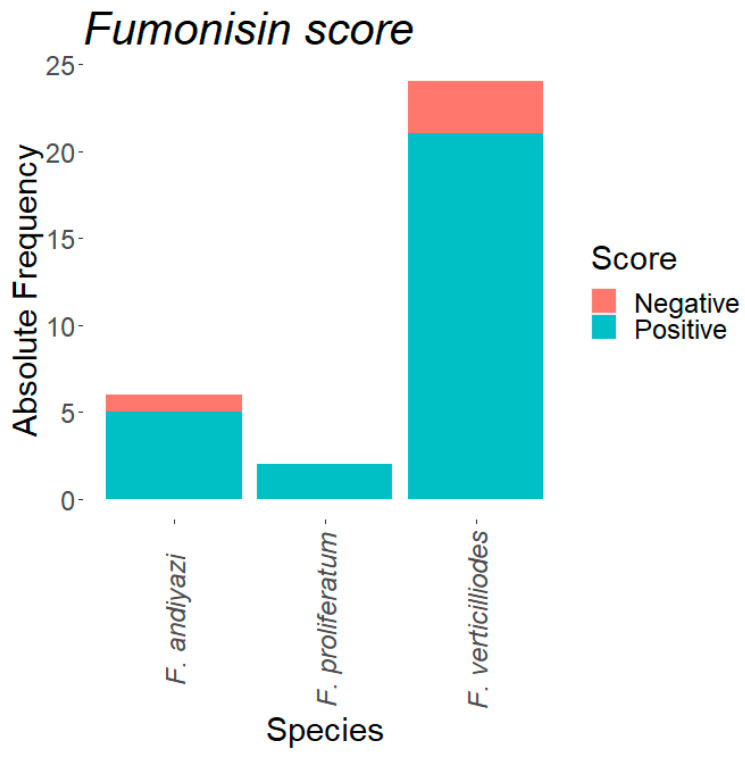
Fumonisins production capabilities among *Fusarium* species isolated from northern Uganda.

The majority of the isolates produced a high quantity of fumonisins in growth media. The proportion of *F. verticillioides* (88.46%, *n* = 23) that was able to produce fumonisins was significant (X^2^ = 13.88, *p* = 0.0001), higher than the proportion of *F. verticillioides* that was not able to produce fumonisins (11.53%, *n* = 3). The three isolates not capable of producing fumonisins were UgB08, UgA03, and UgD01. All the *F. proliferatum* 100% (*n* = 2) isolates were capable of producing fumonisins in PDA media. Among the *F. andiyazi* population tested, a greater proportion (87.5%, *n* = 7) of the isolates produced fumonisins in media, and only one isolate of *F. andiyazi* tested did not produce fumonisins in the media (Figure 3).

**Figure 4:**
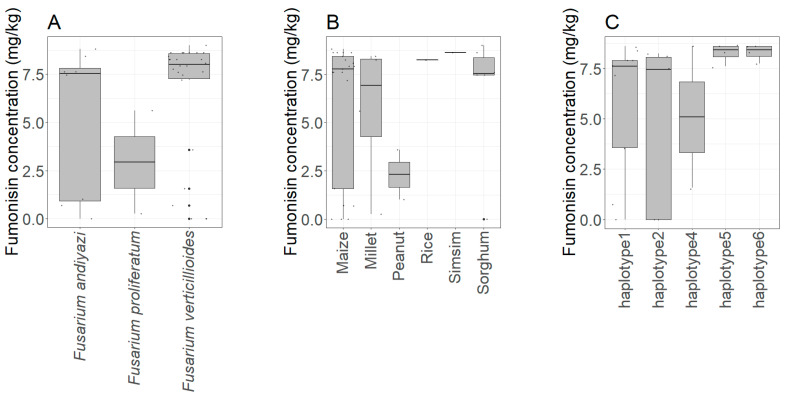
Fumonisins production among three species of *Fusarium* isolated from northern Uganda. (**A**) Variation in concentration of fumonisins among the species. (**B**) Variation in fumonisins production among isolates derived from different grain types. (**C**) Variation in fumonisins production in different haplotype groups of *Fusarium verticillioides*.

Mean fumonisins concentrations produced by *F. andiyazi* and *F. verticillioides* were higher than mean fumonisins concentrations produced by isolates of *F. proliferatum*. However, the difference in the concentration of fumonisins produced by three *Fusarium* species was not statistically significant (X^2^ = 3.24, *p* = 0.20). In terms of the level of fumonisins production, isolate UgB06 of species *F. verticillioides* produced the most fumonisins (9.009 mg/kg). Among the *F. verticillioides* capable of producing fumonisins, three isolates (UgF09, UgA01, and Ug11) produced low amounts of fumonisins (less than 5 mg/kg) whereas the majority of the isolates had a production level exceeding 5 mg/kg. Among *F. andiyazi*, two isolates (UgH01 and UgD09) had fumonisins production levels below 5 mg/kg. The fumonisins production capability of *F. verticillioides* was not dependent on the grain type from which they were isolated (X^2^ = 4.0, *p* = 0.55) (Figure 4). Similarly, the fumonisins production was not dependent on the haplotype group of the *F. verticillioides* isolates as no significant differences (X^2^ = 6.38, *p* = 0.17) in fumonisins concentrations between the five haplotype groups were observed (Figure 4).

## 4. Discussion

To assess the risk of mycotoxin contamination of grains in Uganda (northern Uganda) fungi were isolated from grains collected from different households. A total of 160 isolates were characterized based on both the ITS and TEFα-1 gene region. The result indicates the occurrence of many fungal genera and species on grains intended for human consumption in Uganda. *Fusarium* species were most frequently isolated, followed by fungi belonging to the genera *Curvularia* and *Bipolaris*. Both *Curvularia* and *Bipolaris* are crop pathogens that result in significant crop losses globally every year [37,38]. Although they are regarded as plant pathogens, information on their occurrence and extent of damage to cereal production in Uganda is limited. The extent of crop losses due to *Curvularia* in Uganda might exceed those reported in developed countries given the high prevalence as detected in this study. Both *Bipolaris* and *Curvularia* appeared to be associated with most grains commonly grown (maize, millet, rice, and sorghum) in northern Uganda. This might indicate that many people are at risk of utilizing grains contaminated with *Curvularia* derived mycotoxins such as methyl-5-(hydroxymethyl) furan-2-carboxylate produced by *curvularia lunata* [39] coupled with other mycotoxins such as aflatoxins, which are already known to be common in grain-based food in Uganda. This study, therefore, provides a background for the need to determine dominant *curvularia* species and profile crop losses due to *Curvularia* and *Bipolaris* infection and the occurrence of the *Curvularia* derived toxins in human diets in northern Uganda to improve food safety. The non-amplification of *Curvularia*, *Bipolaris*, and several other fungal genera with TEFα-1 gene primers pairs (EF1 and EF2) need to be further explored.

*Fusarium* species, considered the most destructive pathogens of plants [40,41,42], were the most common fungi occurring in maize, sorghum, millet, peanut, and rice in northern Uganda. Among the *Fusarium* genus, *F. verticillioides* had the highest frequency of occurrence. This species was isolated from maize, millet, sorghum, and peanut, underscoring its broad host range as reported in other studies [43]. Among the diseases caused by *F. verticillioides*, ear rots and stalk rot in maize, sorghum, and millet [44], and root rot in peanut [45], are the most important. The frequent occurrence of *F. verticillioides* highlights the burden of the diseases in grain production among farmers in Uganda. The fungi were isolated from household grains that are sometimes used as seeds by the majority of farmers according to traditional means of seed acquisition. Such grains, which are contaminated with *Fusarium*, may play a critical role in the spread of fungi to new cereal fields among local farmers when used as seeds [46]. The use of clean grains would help to minimize the perpetuation and spread of *Fusarium*. Besides the spread of *Fusarium* by seeds, some *Fusarium* strains can survive for years on crop debris left in the fields and become an important source of inoculum to new crops in the following growth season [47]. Managing crop residues and use of clean seeds is a very important approach in the control of *Fusarium* infections and their associated mycotoxins. Practices such as burning or burying crop residues by deep ploughing during land preparation would reduce Fusarium inoculum for the subsequent crop [48]. Furthermore, irrigation, crop rotation farming, and use of recommended fertilizer were shown to lower the damage associated with *Fusarium* fungi in the field [49]. The high occurrence of *Fusarium* among household grains is an indication of a deficit in the implementation of best practices that reduced *Fusarium* load in the field among farmers of northern Uganda. Despite the low occurrence of *Aspergillus flavus* among the isolates analyzed, aflatoxin has been demonstrated to be common among grains in Uganda and is considered a major mycotoxin that consistently contaminates grains in Uganda [13,19].

The *Fusarium* species that were isolated from Uganda were not closely related to species that were reported in other countries when compared using TEF-α1 partial gene sequence. In particular, *F. verticillioides* isolates derived from Europe, Japan, and Australia showed high genetic differentiation with isolates derived from Uganda. This finding suggests that Europe and other countries have limited gene flow with Uganda and might offer little threat of introducing *Fusarium* strains into Uganda’s cereal production system. Contrary to what was observed for other countries, our results indicate low genetic differentiation between isolates of *F. verticillioides* from Uganda and isolates reported in China within the TEF-α1 gene. The reason for this close relationship between the *F. verticillioides* from Uganda and China is not clear, and probably the use of many gene loci would give a better understanding of the relationship between isolates from the two countries. We also noticed moderate genetic differentiation between Ugandan isolates (collected in 1997) from eastern Uganda [34] and isolates from northern Uganda (collected in 2018) for this study, which could be due to temporal lapse and differences in the agro-ecological zone.

For Uganda, the *F. verticillioides* population groups into five different haplotype groups based on the TEF-α1 gene, which confirmed earlier reports that indicate five haplotype groups in Uganda [34]. We can now deduce that there are five lineages or phylogroups of *F. verticillioides* associated with cereal grains in Uganda as shown in this and previous studies base on TEF-α1 gene. Based on the TEF-α1 sequences from *F. verticillioides* derived from many countries; they grouped into many haplotypes and some haplotype groups appeared to be localized only in some countries, while other haplotype groups occurred in many countries. For example, haplotype 6 seemed to occur only in Uganda, whereas haplotype 1 occurs in many countries with the highest frequency relative to other haplotype groups. Haplotype 1 appears to be the dominant haplotype of *F. verticillioides*, adapted to survive in a broad range of climates, but some underwent local diversification in different geographical areas to give rise to other haplotype groups that now show limited geographical dispersion. Ugandan populations of *F. andiyazi* had the highest intra-species diversity and divergence from populations in other countries, which correlate to what was observed with genetic differentiation between Ugandan isolates and those reported elsewhere. The *F. andiyazi* isolates from Uganda cluster into eight haplotypes representing the most diverse *Fusarium* species associated with grains in Uganda. None of the Ugandan isolates belong to haplotype groups detected in other countries, whereas haplotype 9 of *F. andiyazi* appeared to be the dominant group occurring in many countries excluding Uganda. The limited occurrence of Ugandan haplotypes elsewhere show a limited interaction of Ugandan isolates of *F. andiyazi* with other countries and suggests that the genetic structure and diversity within Uganda is largely shaped by selection pressure in the farming atmosphere relative to gene flow. The ubiquitous *F. equiseti* isolates were divided only into three haplotype groups and appear to be less diverse within TEF-α1 gene compared to isolates reported in other geographical areas [50]. The dominant group of *F. equiseti* in northern Uganda are those belonging to haplotype 22. The three isolates of *F.equiseti* (UgF02, UgF11, and UgG02) with a unique variant of the nucleotides contrary to the dominant haplotype 22 group could represent different lineages that are marginally occurring within Uganda. As well, the *F. incarnatum* isolates in Uganda were less diverse, with only three haplotype groups just like *F.equiseti*. Generally, *F. verticillioides’* haplotype groups detected in Uganda show wider geographical distribution in contrast to what was observed for the species of *F. andiyazi* and *F. equiseti*, in which all the haplotypes detected in Uganda were found not to occur in other countries. On the other hand, *F.equiseti* and *F. incarnatum* associated with grains in Uganda appear to be less diverse, as both showed a limited number of haplotype groups compared to *F. andiyazi* and *F. verticillioides*, which show high diversity with a relatively high number of haplotype groups.

The ability of the *Fusarium* species to produce fumonisins was analyzed by identifying two genes involved in the fumonisin metabolic pathway (FUM1 and FUM3) [51]. The occurrence of the fumonisins gene cluster in *F. verticillioides* isolates was high compared to the occurrence in *F. andiyazi*, which suggests that *F. verticillioides* could be the major fungus associated with fumonisins contamination of grains in northern Uganda. The non-detection of *FUM1* and *FUM3* in some isolates might be due to a mutation that alters the open reading frame of the gene [52,53] or due to a deletion of the gene. Such differences can be revealed through high-throughput sequencing or long-read sequencing that cover the entire gene. Detection of the fumonisins gene cluster was validated by quantification of fumonisins production of the isolates on potato dextrose agar. The findings indicate that the majority of isolates of *F. verticillioides*, *F. andiyazi*, and *F. proliferatum* isolated from northern Uganda were able to produce fumonisins. The fumonisins production did not depend on the *F. verticillioides* haplotype nor on the isolation source, which is in contrast to what was observed in other countries [54]. This implies that all the cereal grains are highly susceptible to fumonisins contamination and that all haplotype groups are equally potent fumonisins producers. As well, no significant differences in the concentration of fumonisins produced by the three species of *Fusarium* were observed, which implies the various species present a similar magnitude of threat in regard to food safety in northern Uganda and other places that rely on cereal grains produced from the area. The high prevalence of fumonisins producing isolates among household grains may correlate to their abundance in the farmers’ crop field which present significant threat in term of crop damage and toxins. The occurrence of the fungi in all the grain types means that they are widely spread among staple grains produced in Uganda. As the majority of the people rely on these cereals (maize, millet, and sorghum); it indicates a high level of exposure to fumonisins among people in this area through dietary consumption of grains. As the isolates produce a vast amount of fumonisins within a short period, it might reveal that the majority of grains get contaminated at a level above the recommended tolerable limit of 1 μg/kg.bw [55] given the widespread poor pre- and post-harvest practices that are among the farmers. The contamination of grains with fumonisins not only causes a health burden to the consumers of the grains, but also reduces access to cereal grains to better markets, which exacerbates poverty among the people. The high incidence of fumonisins producing isolates might be due to their competitive advantage over the atoxigenic variants within the agricultural production area of northern Uganda. Intensive cultivation of staple crops (maize, millet, and sorghum) by the majority of people coupled with poor farming practices supports the proliferation and multiplication of *Fusarium* species. However, the underlying mechanisms supporting the high incidence of toxigenic *Fusarium* isolates need to be further investigated as to whether it comes from poor farming practices or whether it is driven by climate conditions. It has been observed that mycotoxigenic fungi are favored by warm and humid climates [56], which are prevailing in northern Uganda. To control the already high *Fusarium* contamination of grains observed in northern Uganda; crop rotation, deep ploughing, crop residue management, and timely harvest are some management strategies that appear to be affordable to resource-constrained farmers, and they must be encouraged to adopt these practices.

Interspecies polymorphisms of the fumonisins biosynthesis genes show a less divergence of those genes between the *F. verticillioides* and *F. andiyazi* compared to *F. proliferatum*, which correlates with the low nucleotide diversity between the two species based on the TEFα1 gene. Amplification and sequencing of FUM1 and FUM3 gene clusters offer an alternative that can be used in the identification, and discrimination of *F. verticillioides* and *F. proliferatum* in addition to the established TEF1-α method.

## 5. Conclusions

The predominant fungal genus found in northern Uganda was *Fusarium*, and within this genus, *F. verticillioides* was the most frequent species. Most of the grain types were infected with *Fusarium* isolates capable of producing high amounts of fumonisins, which lower the quality of grains used as food in Uganda and consequently, presenting a health burden. Moreover, this study provides evidence that genetic differentiation between the isolates from Uganda and those from other countries is very high, and there seems to be no genetic movement. However, we found a novel haplotype that showed restricted geographical distribution within Uganda only. The frequent occurrence of genes associated with mycotoxin indicates that the majority of the isolates in northern Uganda are capable of producing mycotoxins. The high occurrence of mycotoxin producing isolates is an indication of the contamination of grains with fumonisins and also the potential presence of other mycotoxins that were not evaluated.

## Figures and Tables

**Table 1:** Fungal genera and species isolated from grains in northern Uganda (*n* = 160).

Genus Classification Based on ITS Region	N	Percentage (%)
*Aspergillus*	1	0.63
*Bipolaris*	18	11.25
*Cladosporium*	2	1.25
*Curvularia*	13	8.13
*Diaporthe*	1	0.63
*Epicoccum*	12	7.50
*Exserohilum*	1	0.63
*Fusarium*	85	51.25
*Lasiodiplodia*	8	5.00
*Macrophomina*	3	1.88
*Nigrospora*	1	0.63
*Periconia*	1	0.63
*Sarocladium*	2	1.25
*Trametes*	2	1.25
*Trichoderma*	1	0.63
**Species classification based on TEF1-α**		
*Aspergillus spp.*	1	0.63
*Fusarium andiyazi*	18	11.25
*Fusarium equiseti*	11	6.88
*Fusarium incarnatum*	21	13.13
*Fusarium proliferatum*	2	1.25
*Fusarium solani*	4	2.50
*Fusarium thapsinum*	2	1.25
*Fusarium verticillioides*	32	20.00
*Trichoderma gamsii*	1	0.63
No sequence	4	2.50

**Table 2:** Distribution of fungal species among the grain types using TEF1-α.

Fungus	Maize (*n* = 44)	Millet (*n* = 12)	Peanut (*n* = 11)	Rice (*n* = 5)	Simsim (*n* = 2)	Sorghum (*n* = 22)
*Fusarium andiyazi*	7.50	1.87	0.63	0.00	0.00	1.25
*Fusarium equiseti*	1.25	0.00	3.75	0.00	0.00	1.87
*Fusarium incarnatum*	3.13	3.13	0.00	1.87	0.00	5.00
*Fusarium proliferatum*	0.00	1.24	0.00	0.00	0.00	0.00
*Fusarium solani*	0.63	0.00	1.87	0.00	0.00	0.00
*Fusarium thapsinum*	0.00	0.00	0.00	0.00	0.63	0.63
*Fusarium verticillioides*	13.13	1.25	0.63	1.25	0.63	3.13
*Trichoderma gamsii*	0.63	0.00	0.00	0.00	0.00	0.00

**Table 3:** Genetic differentiation between isolates of *Fusarium* species isolated from Uganda and isolates reported in other countries using partial TEF1-α sequence.

Fungal Species	Country of Origin	Fst	Chi-Square	*p*-Value
*Fusarium verticillioides*	Kenya (*n* = 10)	0.13	19.87	0.00
	Eastern Uganda (*n* = 11)	0.11	10.30	0.07
	china (*n* = 13)	0.03	20.75	0.02
	USA (*n* = 14)	0.12	23.75	0.00
	Italy (*n* = 7)	0.30	6.96	0.14
	India (*n* = 19)	0.21	38.93	0.00
	Argentina (*n* = 10)	0.04	9.51	0.09
	Japan (*n* = 11)	0.43	29.67	0.00
	Spain (*n* = 17)	0.01	12.20	0.09
*Fusarium andiyazi*	Argentina (*n* = 21)	0.33	34.00	0.00
	USA (*n* = 6)	0.24	19.00	0.04
	China (*n* = 19)	0.29	32.00	0.00
	Australia	0.32	24.00	0.01
	Nigeria (*n* = 5)	0.16	18.00	0.02
	India (*n* = 4)	0.05	17.00	0.07
	Brazil (*n* = 9)	0.10	18.55	0.07
	Korea (*n* = 5)	0.35	18.00	0.04
	Italy (*n* = 5)	0.36	18.00	0.06
*Fusarium equiseti*	USA (*n* = 10)	0.36	20.00	0.01
	Italy (*n* = 12)	0.26	22.00	0.04
	Korea (*n* = 3)	0.18	13.00	0.02
	China (*n* = 13)	0.38	23.00	0.00
*Fusarium incarnatum*	Spain (*n* = 5)	0.19	24.00	0.00
	Australia (*n* = 10)	0.47	29.00	0.00
	India (*n* = 8)	0.77	27.00	0.00
	USA (*n* = 4)	0.51	28.00	0.00
	Brazil (*n* = 5)	0.33	24.00	0.00
	China (*n* = 15)	0.64	34.00	0.00

The number of isolates derived from northern Uganda for the four species used in genetic differentiation studies was *Fusarium verticillioides* (*n* = 27), *Fusarium andiyazi* (*n* = 13), *Fusarium equiseti* (*n* = 10), and *Fusarium incarnatum* (*n* = 19).

**Table 4:** Occurrence of fumonisin gene cluster (FUM1 and FUM3) among three species of *Fusarium*.

	*Fusarium* Species	N	Absent (%)	Present (%)
FUM1 gene	*Fusarium andiyazi*	18	38.89	61.11
	*Fusarium proliferatum*	2	0.00	100.00
	*Fusarium verticillioides*	32	3.13	96.88
FUM3 gene	*Fusarium andiyazi*	18	50.00	50.00
	*Fusarium proliferatum*	2	0.00	100.00
	*Fusarium verticillioides*	32	3.13	96.88

## Data Availability

All the nucleotide fasta sequences were submitted to the NCBI database (www.ncbi.nlm.nih.gov) and can be retrieve through the accession numbers found in excel files attached to this manuscript.

## Supplementary Materials

The following are available online at https://www.mdpi.com/2076-2607/9/2/383/s1.
