# Supplementary material for: Genetic Characterization of Fungal Biodiversity in Storage Grains: Towards Enhancing Food Safety in Northern Uganda
Source: Microorganisms. 2021 Feb 14;9(2):383. doi: 10.3390/microorganisms9020383 (PMC7917641; doi:10.3390/microorganisms9020383)
Supplement: Supplementary file 1 [file microorganisms-09-00383-s001.zip › supplementary/FigureS3.pdf]

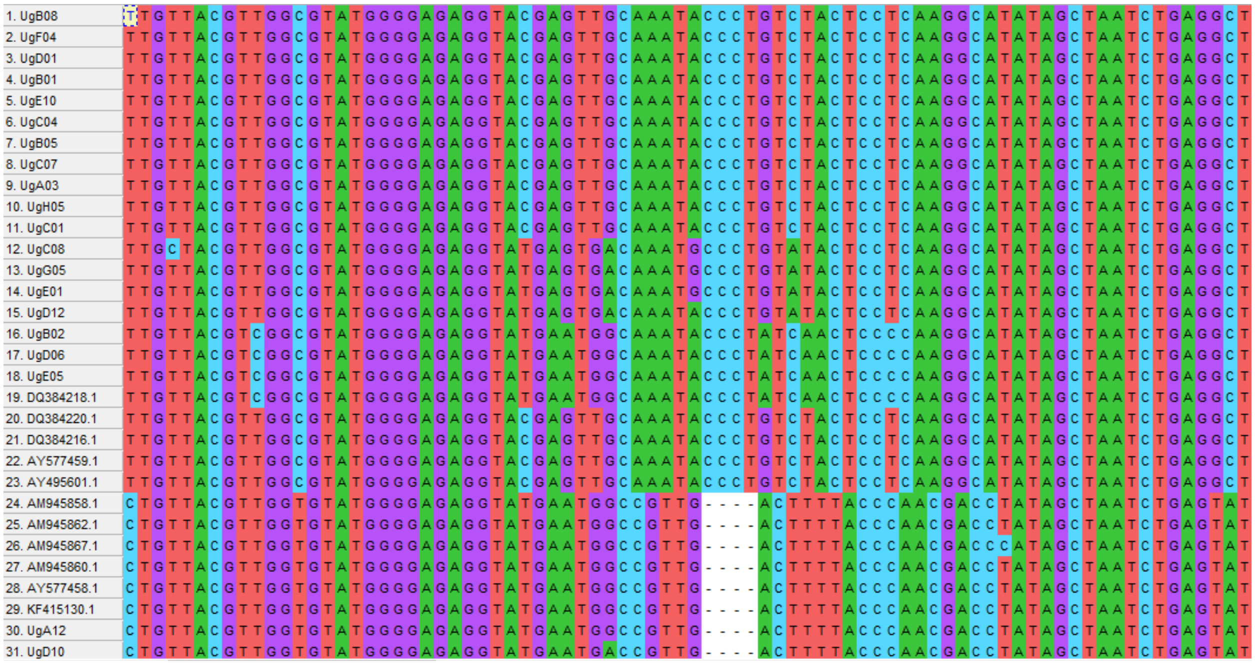
1. UgB08
2. UgF04
3. UgD01
4. UgB01
5. UgE10
6. UgC04
7. UgB05
8. UgC07
9. UgA03
10. UgH05
11. UgC01
12. UgC08
13. UgG05
14. UgE01
15. UgD12
16. UgB02
17. UgD06
18. UgE05
19. DQ384218.1
20. DQ384220.1
21. DQ384216.1
22. AY577459.1
23. AY495601.1
24. AM945858.1
25. AM945862.1
26. AM945867.1
27. AM945860.1
28. AY577458.1
29. KF415130.1
30. UgA12
31. UgD10
