# Supplementary figures and images for: Genetic Characterization of Fungal Biodiversity in Storage Grains: Towards Enhancing Food Safety in Northern Uganda
Source: Microorganisms. 2021 Feb 14;9(2):383. doi: 10.3390/microorganisms9020383 (PMC7917641; doi:10.3390/microorganisms9020383)

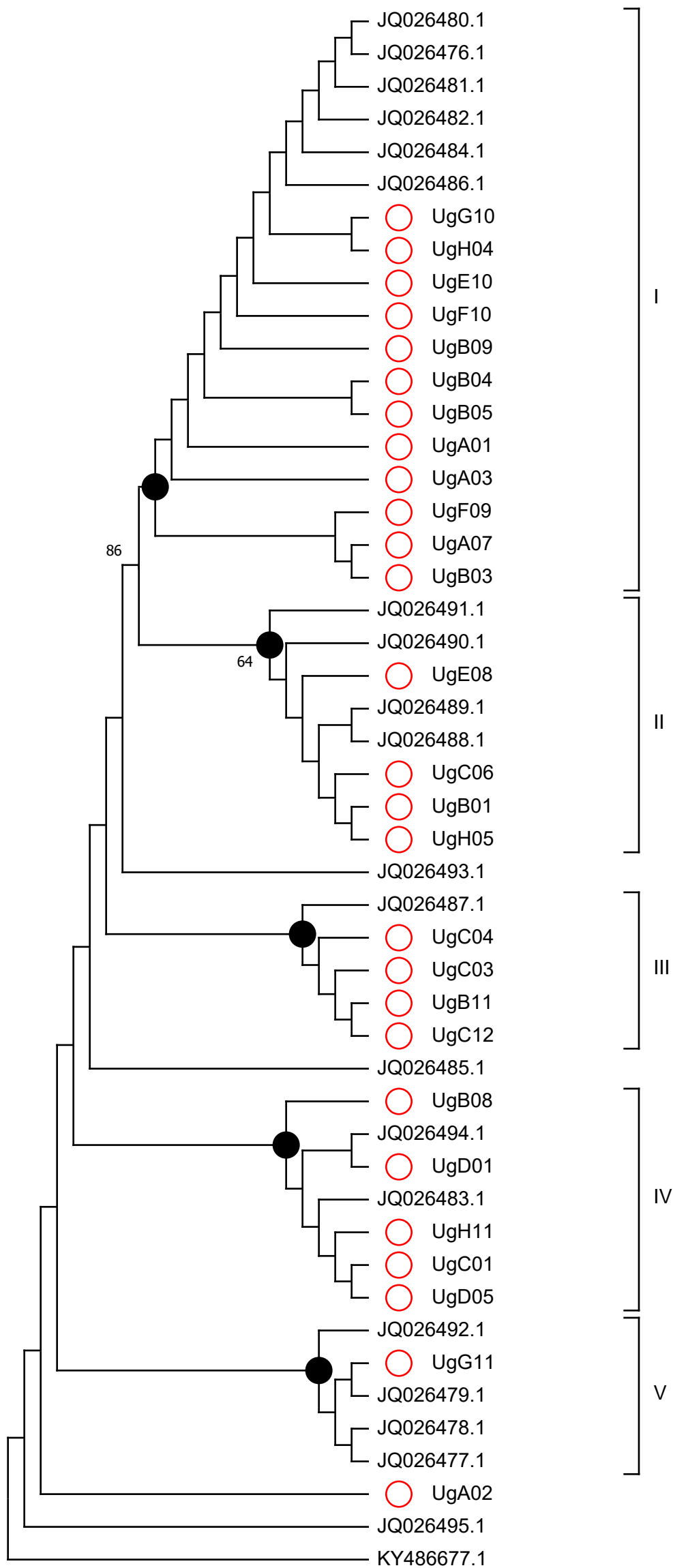

Supplement: Supplementary file 1 [file microorganisms-09-00383-s001.zip › supplementary/FigureS1.pdf]

*F. verticillioides*

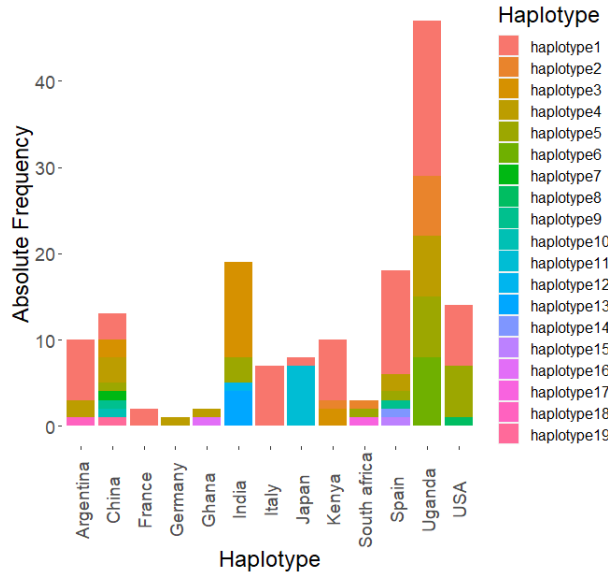

*F. andiyazi*

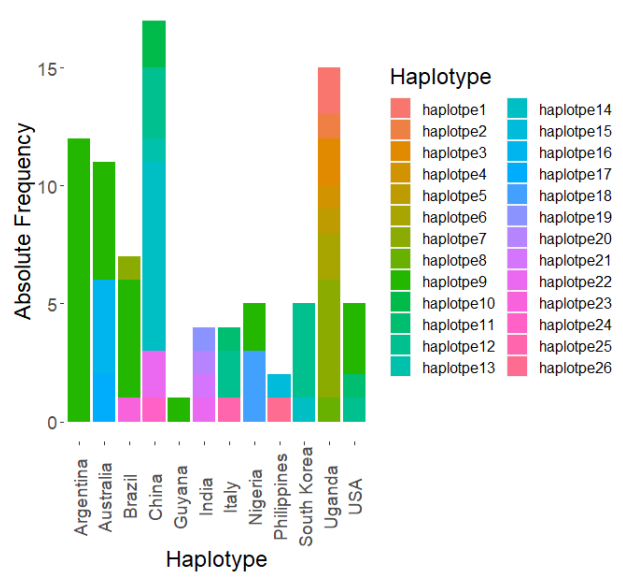

*F. equiseti*

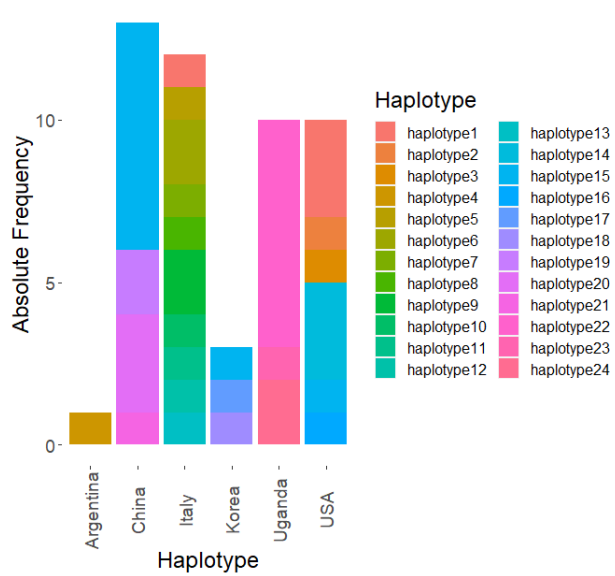

*F. incarnatum*

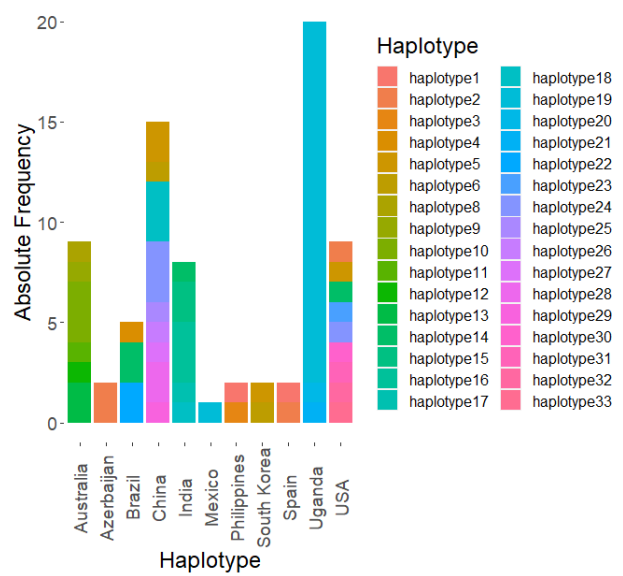

Supplement: Supplementary file 1 [file microorganisms-09-00383-s001.zip › supplementary/FigureS2.pdf]
